# Technological Solutions for Social Isolation Monitoring of the Elderly: A Survey of Selected Projects from Academia and Industry

**DOI:** 10.3390/s22228802

**Published:** 2022-11-14

**Authors:** Ghazi Bouaziz, Damien Brulin, Eric Campo

**Affiliations:** LAAS-CNRS, Université de Toulouse, CNRS, UPS, UT2J, 31400 Toulouse, France

**Keywords:** elderly monitoring, social isolation, identification of ADLs, smart home, health monitoring

## Abstract

Social isolation is likely to be one of the most serious health outcomes for the elderly due to the COVID-19 pandemic, especially for seniors living alone at home. In fact, two approaches have been used to assess social isolation. The first is a self-reported survey designed for research purposes. The second approach is the use of monitoring technology. The objective of this paper is to provide some illustrative publications, works and examples of the current status and future prospects in the field of monitoring systems that focused on two main activities of daily living: meal-taking activity (shopping, cooking, eating and washing dishes) and mobility (inside the home and the act of going out). These two activities combined seem relevant to a potential risk of social isolation in the elderly. Although current research focuses on identifying only ADLs, we propose to use them as a first step to extract daily habits and risk level of social isolation. Moreover, since activity recognition is a recent field, we raise specific problems as well as needed contributions and we propose directions and research opportunities to accelerate advances in this field.

## 1. Introduction

Human beings are fundamentally social creatures who cannot survive without depending on each other. High-quality social connections are essential to our well-being. However, social isolation is widespread and can affect people, especially the elderly who are the most vulnerable population to this problem. It was estimated that approximately 12% of seniors feel socially isolated, according to data from the Canadian Community Health Survey—Healthy Aging 2008/09 [1]. Social isolation among seniors is therefore a growing concern, as the COVID-19 pandemic and accompanying physical distancing measures have increased the importance of these topics.

Social isolation can be defined structurally as the absence of social interactions, contacts, and relationships with family, friends, neighbors on an individual level, and with society at large on a broader level [2]. Furthermore, the term social isolation is often conflated with loneliness but represents a distinct concept. Social isolation is the objective state of having few social relationships or infrequent social contact with others, and loneliness is a subjective feeling of being isolated [3].

Social isolation of the elderly is related to distinct reasons such as age, gender, loss of one’s partner, lack of relationships with family members, friends and neighbors, medical problems, disabilities, rural or urban environment, accessibility to public transport, accessibility to all daily services, low income, low knowledge of modern technologies, heatwave period, infectious diseases, etc.

Social isolation has different impacts on the elderly:

- Psychological impacts: the lack of contacts can lead to spending several days without speaking to anyone [4] and, as a result, different psychological problems could appear including despair, depression, stress and the lack of self-esteem may result in committing suicide [5].
- Physical impacts: Social isolation limits the elderly’s relationship with the outside world which leads to a decrease in physical activities that are important for their health and well-being. Consequently, it leads to a decline in physical abilities [6].
- Health impacts: Social isolation has devastating effects on the health of the elderly, particularly at the nutritional level. Indeed, the risk of malnutrition have increased among the elderly who are socially isolated [7].

That is why in this paper we focus on identifying potential candidate with risk of social isolation by measuring if there is a behavioral drift in the activities that are done daily by the senior. The activities are meal-taking and mobility activities which are risk factors of social isolation.

The excessive cost and limited number of places in residential care facilities for dependent elderly people, their desire to stay at home, and the huge cost of health care have encouraged the implementation of monitoring, alerting and motivational support systems for home care. It is a solution to promote voluntary home care and prevent social isolation. Aging in place is common among the elderly worldwide. Indeed, 84% of people aged 60 and over in France had expressed their wish to live at home, 45% of them with helpers [8], and 86% of baby boomers/elderly homeowners in major metropolitan areas in Canada wish to live in their current home for as long as possible [9].

The objective of this paper is to review the current state and research in the field of monitoring systems that focus on two activities of daily living: meal-taking activity (shopping, cooking, eating and washing dishes) and mobility (inside the home and the act of going out), which seem relevant to a potential risk of social isolation in the elderly living alone. As this study is not an exhaustive presentation of the scientific literature in the field of monitoring systems focusing on meal-taking activity and mobility, only a few projects from academia and industry are presented.

The paper is organized as follows: Section 2 describes methods to include articles in this review. Section 3 presents current issues concerning monitoring systems. Section 4 presents the monitoring system features. Section 5 presents the hardware and software used in different systems. Section 6 discusses examples of monitoring systems. Section 7 analyses the findings of the review. Finally, Section 8 draws the main conclusion of this work.

## 2. Methods

### 2.1. Overview

The desire to live independently at home is increasing dramatically among the elderly due to attachment to their home and the increasing cost of the care in retirement homes. With increasing advances in monitoring system technology, seniors can stay at home while their families feel safe. Elderly monitoring systems have developed rapidly due to the advances in sensors and devices such as miniaturization, wireless communication capabilities (Wi-Fi, Bluetooth Low Energy, Z-Wave, Zigbee, EnOcean, etc.), reduced power consumption, and affordability. These technological advances allow the elderly monitoring systems to be unobtrusive, non-intrusive, and highly effective.

In this review, specific selection criteria are chosen to reference articles on elderly monitoring systems that focus on two key areas of ADLs, mobility and nutrition, to identify social isolation.

### 2.2. Inclusion Criteria for Elderly Monitoring System Research

There are three approaches to design monitoring systems: (1) wearable sensors such as accelerometer, hand-worn sensors, vital monitoring sensors, etc.; (2) non-wearable sensors such as pyroelectric infrared sensors, ultrasonic sensors, reed switches, pressure sensors, power meters, audio sensors, cameras, etc., and (3) hybrid system consisting of wearable and non-wearable sensors. All these sensors have been used in different studies, but the systems that use the cameras are the least preferred. Despite the fact that these cameras provide a precision in the tasks performed and an accurate representation of the situation, they are considered as a serious privacy violation while monitoring the elderly at home.

Studies were included when: (1) wearable, non-wearable or hybrid system was used for activity recognition; (2) the activities considered are meal-taking activities and mobility; (3) the system able to detect behavioral drift in the ADLs of the elderly that could be linked to health problem such as loneliness, social isolation and malnutrition; (4) they were written in English; (5) they were published after 2010.

We have focused in our study on systems that do not use cameras and have the following characteristics: smart, portable, non-intrusive, wireless, contactless, remote and home-based. We have also focused on providing a minimum of paper per hardware system model to present the different hardware choice used to identify ADLs related to eating activity and mobility.

### 2.3. Research Methods and Strategy

This literature review is not intended to include an exhaustive search of scientific works and publications and does not conduct a systematic review. Rather, this review focuses on the presentation of some illustrative publications, works and examples of hardware and software adopted in the current elderly monitoring systems. We included recent journal publications, conference publications, magazines, information in related websites. The keywords used for the literature search are shown in Table 1.

### 2.4. Results

In our search, we tried to find articles, abstracts and websites with the keywords listed in Table 1. The keywords are used alone or combined using and/or operators. Due to the enormous number of articles and abstracts retrieved, it was decided to include only the articles published for the period 2010 to 2020 in Web of Science, PubMed and IEEE Xplore. Some websites describing projects or reports from governmental or international institutions were included when the published scientific literature did not provide adequate information on the subject. Taking into consideration the number of hits in the bibliographic database for each keyword, we can find the hotspots of research in this field and the aspects that are still rarely covered. As this review is not an exhaustive presentation of the scientific literature in the field of elderly monitoring systems, only a few representative research and development projects or products from academia or industry are presented.

**Figure 1:**
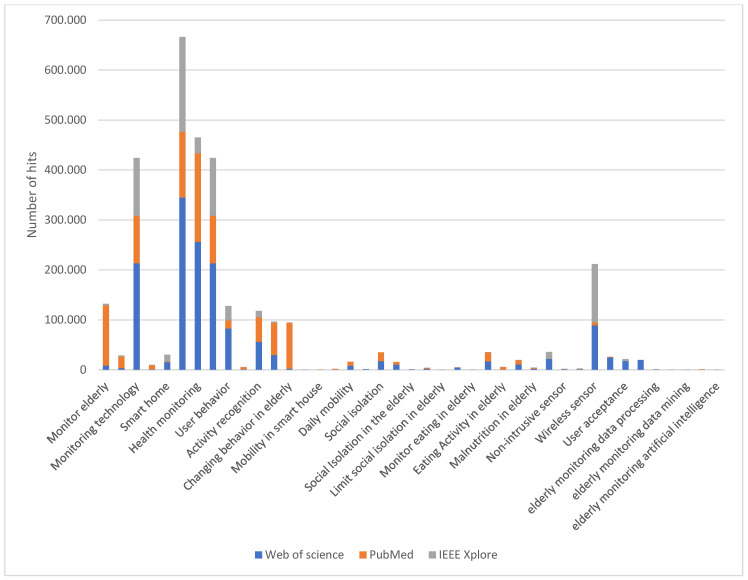
Number of hits in the field of research on elderly monitoring systems between 2010 and 2020.

The number of hits in the elderly monitoring system research area between 2010 and 2020 is shown in Table 2 and Figure 1.

Table 2 and Figure 1 show that the number of hits related to mobility and nutrition activity and the problem of social isolation of the elderly is lower than that of the other keywords. Research in this area is not well addressed, but it is currently becoming very promising for different reasons: (i) advances in technology that are improving the monitoring system; (ii) increasing longevity and aging of the world’s population leads to a greater number of older people in a situation of social isolation; (iii) recent attention towards health effects of social isolation and loneliness received public attention nationally and internationally through mass media coverage, the work of nonprofit organizations and governmental initiatives. For example, in January 2018, the United Kingdom government established and appointed a Minister of Loneliness to develop policies for both measuring and reducing loneliness [10]; (iv) the new impact of COVID-19 on their lives. Pandemic-linked isolation has been blamed for the first uptick in Japanese suicides in 11 years. That is why the Japanese government in 2021 has appointed a Minister of Loneliness to implement policies designed to fight isolation and lower suicide rates [11].

## 3. Current Issues in the Elderly Monitoring Systems

### 3.1. User Needs, Perception and Acceptance

User needs, perception and acceptance are the three factors to take into consideration before designing an elderly monitoring system.

The most important function of an elderly monitoring system is to provide a sense of safety for the elderly themselves and their families, especially for those who live alone. Indeed, the system provides safety by detecting emergencies and alerting caregivers/families such as a fall and/or a decrease in daily activities (meal preparation, daily grooming, etc.). This includes obtaining accurate and complete ADL information. These needs are usually met by the use of a smartphone and various sensors embedded on the body or deployed in the home environment.

The user perception of elderly monitoring system depends on its type (wearable or non-wearable sensor), visibility and privacy. Considering the type, it is preferable to use non-wearable sensors rather than wearable sensors, as they are non-invasive, non-intrusive and contactless. As far as the visibility is concerned, it is important to use miniaturized and wireless sensors and to choose locations that make them quite invisible after a brief period of time. With respect to privacy, the use of cameras and audio recordings is generally not considered as a way to preserve privacy.

A study conducted to obtain older people’s perspectives on the use of sensors [12] indicates that older adults surveyed positively evaluate sensor monitoring because it provides a sense of safety, especially for those who live alone and have therefore experienced a lack of this feeling. Participants also expressed relief that the sensors required no action due to their lack of technical knowledge. In addition, participants did not experience the presence of the sensors in their homes as disruptive. Most reported that they did not notice the sensors after a while. In addition, sensors that record their movements at home without cameras or sound recordings are not considered as an invasion of their privacy.

The willingness of the older person to use the elderly monitoring system is influenced by various factors such as concerns about the technology (technical errors, etc.), positive characteristics of the system (e.g., ease of use factors, privacy implications), expected benefits of the technology such as increased safety, need to use the technology (e.g., perceived need to use), social influence (influence of friends and family), and characteristics of the elderly (e.g., past experiences, physical environment). However, the most mentioned factors were social influence and time to try the technology [13].

### 3.2. Architecture Selection and Requirements

The design of an elderly monitoring system requires the application of several features. The overall architecture of the system must meet the following requirements:

- Heterogeneity: it refers to the fact that IoT systems are composed of different components with different communication protocols, and despite this diversity, they can be integrated into a single system.
- Interoperability: it refers to the ability of the system to provide easy and understandable interfaces by all IoT components and to exchange data between them.
- Maintainability: it refers to the ability of the system to work despite the updates to its components or the addition of new components and therefore to maintain it over time.
- Scalability: it refers to the ability of the system to work as intended despite the changes in the number of users or in the hardware or software.
- Reliability: it refers to the ability of the system to consistently perform as expected and therefore be trusted.
- Efficiency: it refers to the ability of the system to perform in the best feasible way by optimizing time and resources.
- Effectiveness: it refers to the ability of the system to function as intended or to produce the expected results.
- Security: it refers to the ability of the system to ensure the security of data when it is transferred or saved as it relates to the privacy of users.
- Adaptability: it refers to the system’s ability to meet the needs of each senior, as this type of project must be personalized to the individual’s profile.
- Usability: it refers to the ability of the system to be easy to use for the elderly, regardless of their knowledge of technology. In addition, the system should consider having a function for sharing data and notifying caregivers in case of an emergency so that seniors feel safe in their homes.
- Accuracy: it refers to the ability of the system to provide the adapted services to seniors despite the different profiles and requests of each.

The choice of the system architecture has an impact on the satisfaction of these requirements. That is why, among the different infrastructures proposed, middleware is preferred to facilitate the homogenization of the different technologies and to satisfy the prerequisite characteristics [14].

### 3.3. Hardware and Software Considerations

The choice of hardware depends on two main criteria: cost and convenience.

In terms of cost, the price of the system must be affordable for a large number of expected customers. The installation process of the distinct parts of the system should be considered in the choice of materials, because the easier the installation, the less expensive it will be, thus reducing the overall cost of the system. In addition, for parts that rely on disposable batteries, the lower the power consumption, the fewer battery replacement operations and the lower the system cost. An Australian study of 13 people aged 65 years and older found that the cost of purchasing the system and maintaining it at home was a significant concern for the participants [15].

In terms of comfort, user desire is always to reduce maintenance, if possible, because when they buy an electronic system, they think that its reliability will be exceptionally long. In addition, the non-wearable, wireless and miniaturized system will be the right choice; the system that requires no or few interventions is the most needed because of the seniors’ lack of technological knowledge.

Software development depends on two main criteria: effectiveness and efficiency.

The software must be able to operate efficiently at all stages, from data collection and analysis to adaptive response to the detection of any problems. In addition, the software must be efficient in optimizing power consumption especially for sensors that use disposable batteries to send data. Finally, an optimized algorithm is needed to identify ADLs and respond in real time to the detection of a problem.

### 3.4. Ethical Considerations

With the technological advances, sensor-based approaches are now used in clinical practice, research, and to monitor the health of people in homes around the world. Even though technology has its benefits, it cannot neglect the ethical practices. The first concern is the ability of sensors to collect rich information about the lives of older people. For example, a video camera that can identify every ADL in its field of view is considered an invasive sensor. Second, protecting access to research participants’ data is an ongoing privacy concern for all researchers.

This is because anonymity is not always possible. In fact, it can be difficult to manage clinically relevant data to maximize benefits while minimizing the potential for disclosure to third parties. In addition, it is difficult to ensure secure communication during the research process [16]. The third concern is security risk. This is the biggest threat to personal information in the event of a hacking operation, despite the use of encryption software, or if someone accidentally retrieves the participant’s smartphone. A single instance of security breach can negatively impact trust and participation in this type of research [16].

## 4. Monitoring System Features

Automatic ADL classification is a crucial component of assisted living technologies (AAL). It allows monitoring the daily life of older people and detecting any changes in their behavior to encourage them to live independently and safely at home. Many studies on AAL focus on different ADLs, such as bathing, grooming, mobility inside and outside the house, eating, etc. In this study, we will focus on two main ADLs that we hypothesize may be related to social isolation: the process of taking meals (from shopping to dishes) and mobility (inside the home and the act of going out) and their relationship to the social isolation of the elderly.

### 4.1. Meal-Taking Process

The recognition of the activity of eating is particularly important to monitor the health of the elderly. Indeed, nutrition has a significant impact on physical health, memory and mental functions. As we age, good nutrition can boost immunity, fight disease-causing toxins, weight control, and reduce the risk of heart disease, stroke, high blood pressure, type-2 diabetes, bone loss, Alzheimer’s disease and cancer [17]. Unfortunately, undernutrition is common among the elderly and represents a problem that is not yet well studied. For example, the prevalence of undernutrition is estimated in France to be between 3% and 10% for the elderly people staying at home and between 15% and 38% for residents of nursing homes [18]. Identifying all the 12 activities related to eating is the best way to analyze them correctly. The meal-taking process is composed of 4 ADLs: shopping, cooking, eating and dishwashing.

- Food shopping: This is the activity where the person goes to the market to buy different ingredients to cook or to buy already prepared meals. For the elderly, food shopping is not a simple activity, but it is considered an important social event where they can interact with others, as the risk of isolation increases. In fact, for some older people living alone, it is the only opportunity for social interaction. In [19], the authors mention that older people consider the social element and experience of food shopping as a positive factor. The social aspect of food shopping is particularly important to this age group and regular social interaction is recognized as a key element in maintaining mental and physical wellbeing.
- Cooking: The skill or activity of preparing and heating food for eating. Cooking has many physical, emotional, mental and health benefits. This process begins with planning what to cook and what ingredients are needed and if there is a need to go shopping. Then, the person mixes the required ingredients according to a recipe and focuses on the meal until it is properly prepared. This is a good physical and mental exercise. A study of older women in Taiwan found that those who cooked more frequently are engaged in more health-promoting behaviors, such as socializing, and fewer health risk behaviors, such as smoking [20]. In addition, cooking is an opportunity for socialization: seniors can collaborate with each other during meal preparation and sharing food with neighbors and friends is a form of social bonding. Finally, meal preparation allows the seniors to use healthy and fresh ingredients, and thus eat delicious and nutritious meals that they prepared, which they can be proud of. A survey conducted by the University of Michigan National Poll on Healthy Aging in December 2019 shows that many adults between the ages of 50 and 80 reported enjoying cooking (71%) [21].
- Eating: It provides energy to the body. It is important for older adults to stay as active and healthy as possible. Although it is recognized that good nutrition is important for successful aging, malnutrition is one of the greatest threats to the health, autonomy, and well-being of older adults [22]. For the elderly, malnutrition is not the consequence of a lack of food, but of a deterioration in the desire to eat and is related to several factors such as serious health conditions, medication side effects, lack of exercise, difficulties in chewing, swallowing or self-eating, depression, loneliness, and social isolation [23]. Monitoring the eating activity in the elderly is essential to ensuring their well-being.
- Dishwashing: this involves cleaning the dishes of food remains on plates. This can be done manually by hand in the sink or automatically by the dishwasher. Although dishwashing is a light activity, it can be a good physical activity for the elderly that helps prolong their lives. In a U.S. study conducted by the University at Buffalo of more than 6000 white, African American and Hispanic women aged 63 to 99 years, researchers found a significantly lower risk of death for those who were active, even while performing light activity, than in those who were inactive [24].

A use survey conducted by the French National Bureau of Statistics between 2009 and 2010 [25] revealed that the daily time related to the meal-taking activity (shopping, cooking, eating and washing dishes) among people aged 60 years and older is nearly 4 h per day. In fact, men aged 60 years and older spend 24 min shopping, 13 min cooking, 154 min eating and 13 min washing dishes each day. In comparison, women 60 and older spend 21 min shopping, 72 min cooking, 141 min eating and 25 min washing dishes each day. This finding reveals that older men spend less time on household chores than older women, particularly as far as cooking is concerned.

### 4.2. Mobility

Mobility is the ability to move around easily. It can be classified into two types: functional mobility and community mobility. Functional mobility, a basic activity of daily living, is defined as moving from one position or location to another while performing ADLs, such as in-bed mobility, wheelchair mobility, and transfers (e.g., wheelchair, bed, car, bathtub, toilet, tub/shower, chair, floor). It also includes functional ambulation and carrying objects [26]. Community mobility, considered as an instrumental activity of daily living (IADL), is defined as moving around the community and using public or private transportation, such as driving, walking, biking, or accessing and riding in buses, taxi cabs, or other transportation systems [26]. Mobility is important for maintaining self-care and an independent and autonomous lifestyle. In fact, mobility is the key point to performing basic ADLs such as feeding, dressing, toileting and personal hygiene but also the instrumental ADLs such as shopping, preparing meals and cleaning the kitchen after meals.

In addition, regular mobility and activity, even mild physical activity such as walking, improves mental and cardiovascular health, controls weight, maintains healthy bones and muscle strength, reduces the risk of falls and increases social interaction [27].

Furthermore, there is a mobility gap between older men and women. In fact, mobility disability is more frequent in women than in men according to a study done in different places around the world [28].

A U.S. Time–Location Patterns study conducted in six cities indicates that adults aged 65 years and older spend 78% of their time at home, which is high compared to adults aged between 45 and 65 years who spend 66% of their time at home. This result is understandable since seniors are usually retired, have limited social contacts and therefore prefer to spend the majority of their time at home [29]. Therefore, we will focus in our study on tracking mobility inside the home and the act of going out.

### 4.3. Social Isolation and Loneliness

#### 4.3.1. Definitions

Human beings are social animals and our biological, psychological, and social systems have evolved to thrive in collaborative networks of people [30]. Yet, many people suffer from social isolation and loneliness, especially the elderly. While social isolation and loneliness are closely related, they do not mean the same thing. According to the National Institute for Health Research in the United Kingdom, isolation is a lack of social contact or support, whereas loneliness is the feeling of being alone and isolated (it is possible to feel lonely in a room full of people) [31]. A report published by the National Academies of Sciences, Engineering, and Medicine (NASEM) in the United States prior to the COVID-19 epidemic indicated that 24% of community-dwelling adults aged 65 and older in the United States (approximately 7.7 million people) were socially isolated and 43% of Americans aged 60 and older report feeling lonely (approximately 13.7 million people) [32]. With the COVID-19 pandemic, these numbers increased dramatically due to the stay-at-home orders, social distancing, and banning visits for nursing home residents. Social isolation and loneliness are likely to impact the health of the elderly.

#### 4.3.2. Risk Factors for Social Isolation/Loneliness

Older people are vulnerable to social isolation and loneliness due to numerous factors such as living alone, death of the partner, living far from family and friends, living in a rural area, reduced mobility, chronic diseases, digital exclusion, etc. Indeed, the report published by NASEM in the United States indicates that being unmarried, male, having low education and low income is independently associated with social isolation [32]. In addition, the status of social isolation and loneliness depends on gender. Indeed, according to a 2014 report in England [33], 14% of men and 11% of women aged 50 and over experienced a moderate to high degree of social isolation (older men are more isolated than older women); 48% of men and 54% of women aged 50 and over experienced some degree of loneliness (older women are more lonely than older men). In addition, the use of new technologies, especially social networks, has become an important means of communication nowadays. A report conducted by the Pew research center in the USA in 2013 revealed that 27% of all Americans ages 65 and older are on social networking sites. Regarding the use of social networking, older women are more likely than older men to use social networking sites: 52% of female internet users aged 65 and over adopt social networking sites compared to 39% of older men [34].

**Figure 2:**
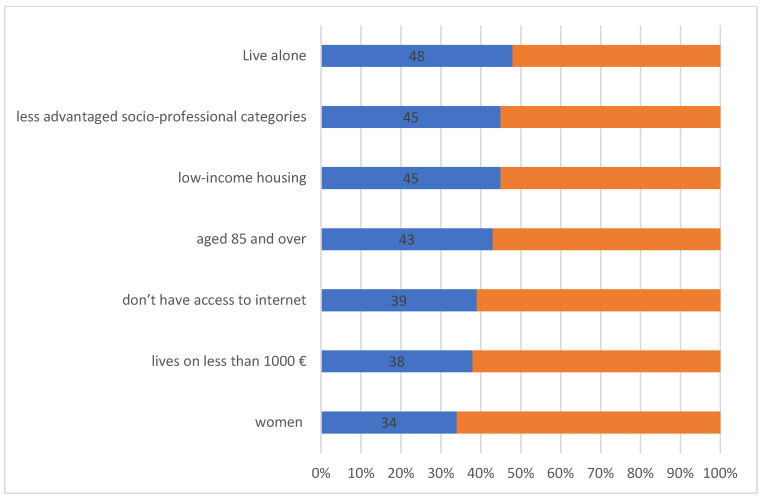
Profile of people aged 60 and over who feel lonely [35].

Furthermore, the French association “Petits Frères des Pauvres” released a report about the links between loneliness, isolation of the elderly, and the territories [35]. It indicates that loneliness is amplified in certain areas and particularly affects elderly over 85 (mainly women) who live alone, belong to less privileged socio-professional categories (with income below 1000€ per month), live in social housing, and have no access to internet. Figure 2 illustrates the percentage of people feeling loneliness for each category.

In addition, there are significant differences in the components of isolation depending on the territory. In urban areas, isolation is worsened by the weakening of solidarity and neighborly relations, the replacement of local shops by shopping malls in the suburbs, the feeling of insecurity, the crowded public transport and its inaccessibility, particularly for people with reduced mobility. For example, 24% of people aged 60 and over living in apartments in France can spend days without talking to anyone (the national average being 19%) [35]. In rural areas, even though solidarity between people is stronger, the lack of public and health services, local shops and public transport, and the fact of losing the autonomy to drive the car due to aging reinforce isolation [35].

Table 3 shows that there is a difference in degree of loneliness (occasional or frequent), with some factors depending on the territory around France.

#### 4.3.3. Social Isolation/Loneliness Evaluation

Different measurement scales have been developed to assess social isolation/loneliness (SI/L) and most of them are self-report questionnaires that were designed for research purposes. The measurement scales developed for the assessment of SI/L are summarized below.

The Berkman–Syme Social Network Index (SNI) is a self-reported questionnaire that measures the level of social isolation. It is a composite measure of four types of social ties: marital status, sociability (number and frequency of contacts with children, close relatives, and close friends), religious group membership, and membership in other community organizations. The SNI scale allows researchers to categorize individuals into four levels of social isolation: socially isolated (individuals with few intimate contacts—unmarried, fewer than six friends or relatives, and no church or community group membership); moderately isolated; moderately integrated, and socially integrated [36,37].

The Lubben Social Network Scale (LSNS) is a 10-item instrument designed to measure social isolation in older adults that addresses the size, closeness and frequency of contacts in the respondent’s social network. Six-item (LSNS-6), twelve-item (LSNS-R) and eighteen-item (LSNS-18) versions of this scale were published after the LSNS. The LSNS was modified to become the LSNS-R to better specify and distinguish the nature of family, friendship and neighborhood social networks. In addition, the LSNS-6 was developed as a short form for clinicians and the LSNS-18 as a long form for research purposes [38,39,40,41].

The Steptoe Social Isolation Index was created by Steptoe and colleagues (2013) to measure social isolation. It is a five-item scale, which focuses on marital status/cohabitation, monthly contact (including face-to-face, by telephone, or written/emailed) with children, other family, and friends, and participation in social clubs, resident groups, religious groups, or committees [42].

The revised UCLA (University of California, Los Angeles) loneliness scale is a commonly used measure of loneliness. It consists of a 20-item questionnaire with four response categories each. A shortened version of this questionnaire, the Three-Item UCLA Loneliness Scale, is being developed for use in telephone surveys [43,44].

The de Jong Gierveld Loneliness Scale is an 11-item self-administered questionnaire for measuring loneliness. It was developed using the Weiss’ (1973) distinction [45] between social and emotional loneliness. It was designed for use with older adults and has been assessed with individuals aged 18 and older. To avoid boredom when using the instrument in large surveys, a short version consisting of 6 items was proposed by the authors. Three statements measure the emotional loneliness and the others focus on the social loneliness, each with three choices: yes, more or less, and no. Focusing on both emotional and social loneliness may provide insight into why a person may experience loneliness [46,47].

There are other different scales which are used to measure the social isolation and loneliness, which were inspired by the above scales. Using validated tools in the assessment of social isolation and loneliness is of the utmost importance. Using an invalidated tool, or just parts of the existing tools, or a tool designed to assess loneliness in a study that is actually examining social isolation may yield inaccurate results.

In addition, technological advances such as machine learning, electronic health records, and predictive analytics hold promise as potential ways to identify social isolation and loneliness. For example, a study using natural language processing techniques to identify mentions of social isolation in clinical notes of prostate cancer patients aged 18 years and older showed satisfactory results in identifying socially isolated patients [48].

Despite the fact that there are different measurement scales developed to assess social isolation/loneliness, they have some limitations. In fact, there are concerns about the quality and appropriateness of current tools, as they were developed decades ago and may not account for new modes of interaction and communication (e.g., social media, instant messaging, video conferencing) [3]. In addition, they are considered as self-report questionnaires and therefore subjective. Furthermore, surveys offer discontinuous observation about the status of the person and therefore cannot detect any problem as social isolation and loneliness may be episodic for some.

#### 4.3.4. Health Impacts of Social Isolation and Loneliness

Increasing evidence demonstrates that social isolation and loneliness are linked to major health risks such as depression, anxiety [49], cardiovascular diseases, mental health problems [50], and death [51]. For example, cumulative data from 70 independent prospective studies with 3,407,134 participants followed for an average of 7 years revealed a significant effect of social isolation and loneliness. After accounting for multiple covariates, the increased probability of death was 26% for self-reported loneliness and 29% for social isolation.

Another health impact of SI/L is decreased mobility. Indeed, any decrease in the physical activity of the elderly has an enormous impact on their autonomy, their ability to live alone at home, and their quality of life. When elderly people suffer from SI/L, they have a limited social network. Consequently, they left their homes less than other people. And with the containment orders due to COVID-19 pandemic, the problem has worsened. This implies muscle loss, decreased physical abilities and fear of falling. The elderly does not want to go out anymore. Thus, they enter a vicious circle where isolation worsens isolation [52]. Furthermore, an English Longitudinal Study on Ageing reveals that older people who experience high levels of loneliness have an increased risk of becoming physically frail [53].

Furthermore, SI/L are major risk factors for malnutrition. Elderly people lose the desire to prepare meals and eat, or they eat little and feel less and less hungry. A study of a total of 1200 randomly selected individuals aged ≥65 years living in rural Lebanon showed that social isolation and loneliness are two independent risk factors for malnutrition in the elderly. The odds of malnutrition were increased by 1.6 in elderly people considered socially isolated and a risk of malnutrition was almost 1.2 times higher in those reporting higher levels of loneliness [7].

The report “ISOLATION OF THE ELDERLY: THE EFFECTS OF CONTAINMENT” by the French association “Petits Frères des Pauvres” indicates that in this period of COVID-19 crisis, many French people have experienced what many elderly people experience all year round, and the fight against isolation is a powerful weapon of prevention [52].

The consequences of social isolation and loneliness for the health of older people will also have an impact on the cost of medical care. Indeed, a recent report in the United States estimates that social isolation of the elderly is associated with an additional $6.7 billion in federal expenditures per year [54].

## 5. Overview of Systems Proposed and Data Collected

Monitoring ADLs of the elderly provides a good overview of their daily routine and health status. It helps to diagnose their ability to live independently and provides an early warning of deteriorating health or early detection of disease.

In this section, we will present a non-exhaustive overview of the different hardware and software propositions.

### 5.1. Hardware Implementation

With the huge technological advances in recent years, several types of sensors have emerged. These sensors are the key to ADL assessment. The choice of sensors depends on several factors such as whether the targeted ADLs are performed inside or outside the house, power consumption, privacy, etc. In this section, we will classify sensors into wearable and non-wearable sensors.

For these reasons, we list the type and position of sensors used in several works, specify the obtained parameter, and briefly analyze the advantages and drawbacks of each type of sensor, as presented in Table 4. Table 5 lists the sensors used in each work. Wearable sensors are attached to the person to collect physiological (temperature, pulse, etc.) and motion (location, step counter, etc.) data. Non-wearable sensors are installed at fixed locations in the home and can collect data on the person’s movements (e.g., position inside the house, opening the outside door to go out), and environment (e.g., humidity, light, temperature inside the home). While non-wearable sensors have the advantage of being autonomous for a long time and do not require user intervention, wearable sensors can be integrated into various objects such as shoes, clothes, patches, etc.

#### 5.1.1. Non-Wearable Sensors

Passive infrared (PIR) is the most used sensor in ADL monitoring studies [51,52,53,54,55]. They can detect motion in specific area and thus the location of the person. They are low cost and easy to install. The PIR sensor can be used in various locations to detect different ADLs such as stove use, toilet use, sleep, etc.

Reed switches are commonly used to detect the opening and closing of the door or cupboard. They can provide information about the use of the refrigerator, medicine cabinet, or the opening of the outer door, etc. [55,56,57,58,59,60,61]. In [62], reed switch sensors were able to detect different activities such as taking medicine, grooming, preparing meals, etc.

Ultrasonic sensors are used in ADL recognition by performing a distance measurement between the sensor and an object. In [63], these sensors installed on the ceiling were used to detect standing, sitting, and falling of a person, as well as movements in different directions.

Video sensors are used to monitor the elderly. They can be installed at various locations in the house and provide rich and detailed information about the monitored person. Analysis of the video recorded by these sensors can identify different ADLs such as cooking, eating meals, drinking, falling, etc. While RGB cameras are standard for the video scene recorded by the researchers [64], this type of sensor is sensitive to light and thus degrades the performance of the ADL recognition. In addition, the captured video contains too much information that may raise privacy issues for the monitored person and lead to their refusal to use it [12]. To solve this issue, researchers proposed the depth camera. This camera captures depth images (containing information about the distance between the corresponding objects and the sensor), which are invariant to light conditions, and the absence of appearance details preserves privacy. In [66], the Kinect was used, which provides color and depth streams to recognize intake actions. Another type of camera to solve the privacy issue is the thermal camera. It creates an image based on the temperature of the corresponding objects. The image does not suffer from light conditions and protects the privacy of the monitored person. In [67], a combination of depth and thermal sensors was used to detect different ADLs such as sitting, walking, sleeping, etc.

The force/pressure sensor is used to provide primary information about the user’s position. These censors are usually installed in beds, sofas, chairs and carpets [56,60]. In [61], pressure sensors were used to detect objects placed on the stove burner to monitor food preparation activity.

Audio sensors are used to detect sounds to recognize ADLs performed by the person. In [68], microphones were deployed at distinct locations in the home. They were able to detect different activities such as dishwashing, meal preparation, and eating.

Flow meters are sensors that detect water usage. They are usually installed on kitchen and bathroom faucets. In [61], flow meters were placed on the kitchen faucets to detect water usage.

Power meters are sensors that can measure the electricity consumption of appliances such as TV, stove, coffee maker, toaster, microwave, etc. Researchers use two types of power meters: a single power meter placed in the main electrical panel of the house and the system that can distinguish the consumption of each appliance, and another power meter attached to each appliance [56,59,61,70,71,72]. They are often used to monitor ADLs by recording the power consumption of an appliance and then translating it into the probability of a particular ADL.

Passive radio-frequency identification (RFID) is often used to identify ADLs. It uses tags attached to objects in daily use and an antenna to extract the location of these objects. With the topological relationships that exist between the physical objects, the system performs the modeling of ADLs. In [73], using passive RFID, the system was able to identify the preparation of a coffee, a sandwich, spaghetti, tea and a bowl of cereal.

#### 5.1.2. Wearable Sensors

The ultrasonic positioning sensor is another type of ultrasonic sensor. It consists of two components: an ultrasound transmitter called a TAG and receivers. The receivers are deployed in specific locations in the house and the ultrasonic transmitter is attached to the person’s body. The system will be able to detect their position inside the house. In [70], different activities such as cooking, taking a meal, washing dishes, watching TV and reading a book were identified using the ultrasonic positioning sensor and power meter.

Active RFID is another type of RFID sensor for ADL monitoring. It uses an RFID reader and several RFID tags attached to various objects to monitor their use by the monitored person. In [65], a wristband containing an RFID reader and various RFID tags were attached to objects including furniture, appliances, and utensils in a smart home. The system was able to identify six ADLs such as walking, sitting and watching TV, preparing cereal, drinking water, preparing utensils and putting them away.

The smartphone contains several types of sensors that can potentially be used for ADL monitoring. It may contain an accelerometer, gyroscope, global positioning system, magnetometer and microphone. All these sensors provide a huge amount of information such as motion, location, phone calls, etc. In [76], the smartphone was used to detect different activities such as hygiene activities, cooking, washing dishes, eating, etc.

Accelerometer is the most widely used wearable sensor for activity recognition. Due to its low price and diffusion in different devices such as smartphones, smartwatches, smart insoles, etc., an accelerometer can detect the fall, awakening, movement and posture of the person [65,74]. In [65], a smart insole with an accelerometer was used to recognize average distance and speed to encourage walking activity in older adults.

Many ADL monitoring researchers use more than one monitoring technology such as PIR with reed switch. Combinations of multiple sensor types also exist, using different wearable sensors, different non-wearable sensors and, finally, wearable and non-wearable sensors. Using distinct types of sensors can enrich the collected information and improve ADL recognition.

Wearable device-based systems are less invasive but not practical in a long-term monitoring application due to their natural flaws such as easy loss of wearable devices, short battery life, constant maintenance, and discomfort of wearing. In fact, a survey of 6223 U.S. adults found that one in ten consumers aged 18 and older own a modern activity tracking device. More than half of those surveyed said that they no longer use their activity tracker, and one-third stopped using the device within six months of receiving it [77]. Finally, systems based on anonymous binary sensors are the most preferable solution for long-term monitoring application, as they do not require any device and do not violate privacy.

As shown in Table 5, the combination of a PIR and a reed switch sensor is most commonly used in this research field, typically a PIR sensor in each room and a reed switch on the exterior door and other elements of the house. In addition, different systems add other types of sensors (pressure sensor, wattmeter, flowmeters, etc.) to the PIR and reed switch sensors in order to extract additional information such as water and electricity consumption, presence of a person in specific place such as sofa improving the identification of some ADLs. With additional information, for example, from the wattmeter, we can identify that the person is watching their TV. However, this comes at the cost of increased installation effort, the price of the entire system and complicates the deployment process. Additionally, the microphone and video camera are still used to identify ADLs despite the privacy concerns. Despite the fact that there are two approaches of using wearable and non-wearable sensor, some researchers use a combination of both, as in the example of Ueda et al. [70] who used the ultrasonic positioning sensors attached to his body and power meters attached to the TV and stove.

### 5.2. Software and Algorithm Processing

Data are collected from sensors previously presented and transmitted to a data processor through a communication medium. Communication plays a crucial role in connecting all of the components such as sensors, gateway, storage hardware and actuators. Many communication technologies and protocols have been used in the smart home, such as Bluetooth Low Energy, ZigBee, Z-Wave, EnOcean and Wi-Fi for wireless communication, European Installation Bus and X10 for wired communication and KNX and Insteon for heterogeneous communication. The next step is data processing, which consists of applying different data processing methods to analyze the collected data: recognizing ADLs, mining behavioral patterns, detecting abnormal behavior, etc. Researchers in the AAL field have used different algorithms to identify ADLs. Table 6 presents the algorithms and results illustrated in several papers.

As shown in Table 6, several types of algorithms have been used to identify ADLs. The most used methods are supervised learning algorithms such as SVM and KNN. Other methods have also been implemented, such as logical method (associating a set of possible activities with the room where the activity is usually performed, etc.), statistical method (C4.5 algorithm), unsupervised learning algorithms (K-means), artificial neural network (DCNN, Pattern Recognition Neural Network, Long Short-Term Memory, etc.) and fuzzy logic. The variety of the used methods provides satisfactory results in the identification of ADLs. In fact, some papers compare the performance of several methods in their work to find the best one [69].

After data processing, the major step is to use the results to empower the resident, family members and caregivers with the smart home system. Human interfaces can be used for different purposes: allowing family members or caregivers to monitor the elderly’s condition, detect abnormalities in activities and send alerts in case of emergencies, remind the resident of scheduled activities, motivate and assist them in activities such as meal preparation, taking medication, etc.

### 5.3. Participants, Duration and Location of Data Collection

Understanding which group of subjects is involved in the research, how long the data are collected, and where the tests are performed is especially important. Indeed, knowing whether the participants are young or old, heterogeneous or homogeneous, the data are collected for a long or short period of time (no data collected for a few days, for example), the data are collected at the home of the elderly where they typically live or in a laboratory smart home can lead to identifying the benefits and drawbacks of each proposed system in terms of hardware and enhancing the robustness of the proposed monitoring system in terms of software.

Unfortunately, not all selected articles reported participant demographics; only eight articles reported this information. As shown in Table 7, five studies recruited people older than 65 in the experiments, and three studies recruited adults aged 20 to 57. The studies involved healthy participants with a few studies involving participants with health problems [59,60,61]. The number of participants varied from 1 participant in [56,58,60,71,72,73,76] to 43 participants in [55]. Not all studies specified the gender of participants. Only nine articles indicate the gender of the participants as shown in Table 7. The duration of data collection varied from a few samples collected for a few hours in [63,64,65,66,68,71,72,73,74,75] to 2 years in [59]. Data were collected in different types of locations, such as participants’ apartments [55,60,62,68,69,74,76], seniors’ living facilities [57], assisted living facilities [61], and laboratory smart homes [56,58,59,63,64,65,66,70,71,72,73,76].

As shown in Table 7, few studies focus on collecting data in the real environment of the elderly over a long period of time. Therefore, more data collection using these conditions is needed to improve current research.

## 6. Monitoring System Examples

### 6.1. Research Prototypes

#### 6.1.1. Binary Sensor Approach

Huynh et al. [55] propose a system composed of two types of binary sensors: (1) a passive infrared (PIR) sensor in each room and (2) a reed switch attached to the main door to detect loneliness and depression of elderly people living at home. After a real deployment of the system in 50 apartments of seniors living alone, the analysis of sensor data was carried out over 6 months to monitor home mobility and outing behavior. In addition, the 11-item version of the Loneliness Scale developed by De Jong Gierveld was used to assess the participants’ social loneliness scale and emotional loneliness scale. The 15-item version of the Geriatric Depression Scale was used to measure depression. The survey data confirm that older adults living alone are at considerable risk of loneliness and depression. Furthermore, the study demonstrates that the system can determine potential candidates with severe loneliness and depression based on the ratio of time spent inside and outside the flat. Experimental results show potential elderly candidates with severe and moderate depression or loneliness issues with an accuracy of 10/16 and a sensitivity of 10/12.

Gochoo et al. [58] propose a deep learning classification method for elderly activities. In particular, Deep Convolutional Neural Network (DCNN) classification approach was used to detect four ADLs: bed to toilet movement, eating, meal preparation, and relaxing. They used an open annotated dataset provided by the Center for Advanced Studies in Adaptive Systems (CASAS) project at Washington State University. Thirty-three PIR sensors and four reed switches were placed at strategic locations to monitor the ADLs of the elderly. They contain monitored data of a cognitively normal elderly resident from the period of 21 months. The algorithm converts the binary sensor annotations into a binary activity image for the four activities. Then, the activity images are used for training and testing the DCNN classifier. Finally, the classifiers are evaluated by the 10-fold cross-validation method. The experimental results show that the DCNN classifier gives 99.36% of average accuracy.

Pirzada et al. [62] propose a system that can identify and predict problems by monitoring the residents’ ADLs. The project used the Massachusetts Institute of Technology dataset collected using more than 80 reed switches installed in two single-person apartments over a two-week period. The sensors were installed on daily activity items such as the cupboard, coffee maker, fridge, etc. Annotated ADLs such as meal preparation and going to work were used as inputs to the algorithm. Then, the K-Nearest Neighbors algorithm (KNN) was used to detect any irregular activity, and as a result, the system generated alerts and sent a message or a call to the family member/caretaker. An interactive user interface was developed to display the user account details, notifications, user statistics and personal details.

Dawadi et al. [59] propose a Clinical Assessment approach using Activity Behavior (CAAB) to model the daily behavior of a smart home resident and predict corresponding clinical scores. The data used are collected from 18 smart homes with residents with a mean age of 84.71 years for 2 years. The homes were equipped with different motion and door contact sensors. They focus on different activities: sleeping, going to the toilet, cooking, eating, relaxing, personal hygiene and mobility of the resident inside the house. Monitored activity is recognized with 95% accuracy based on 3-fold cross-validation. In addition, bi-annual clinical testing was performed by the residents. Tests included the Timed Up and Go mobility measure (TUG), which identifies and characterizes cognitive decline in older adults, and the Repeatable Battery for the Assessment of Neuropsychological Status (RBANS), which measures mobility using a timed task. Participants get up from a chair, walk 10 feet, turn around, walk back and sit down. Next, the CAAB uses statistical features that describe the resident’s daily activity performance to train machine learning algorithms that predict the clinical scores. The CAAB-predicted clinical scores were calculated using the Support Vector Regression (SVR) algorithm. Finally, statistically significant correlations between CAAB-predicted scores and clinician-provided RBANS and TUG scores were found, and this result suggests that clinical score prediction is possible using ADL data collected by binary sensors.

Rebeen et al. [69] propose a method that uses multiple incremental Fuzzy Temporal Windows (FTWs) for feature extraction. In fact, it delays the recognition process to include some sensor activations that occur after the time where the decision has to be made. An evaluation of the method was done with CNN, LSTM and a hybrid model CNN LSTM. Two other extraction features were applied to the data: Long-Short Term Memory (LSTM) and CNN with Equal Size (1 min) Temporal Windows (ESTWs), C4.5 and SVM with Raw Last sensor Activation (RAW) in one-minute windows. The system used three types of binary sensors to collect data for real daily living activities: PIR sensors at distinct locations in the house, reed switches for open/close states of doors and cupboards, and float sensors to measure flushing. The experimental results show better results in ADL identification when recognition of the activity is delayed, preceding 1-min sensor activations with 5-min delays (20-min delay, 1-h delay, etc.) compared to only considering only 1-min delay sensor data. The f1-score result of the CNN LSTM algorithm is equal to 96.97% when there is a 4-h delay in data processing versus 92.49% when there is a 5-min delay.

#### 6.1.2. Binary and Non-Binary Sensor Approach

Kenfack Ngankam et al. [60] present a Night Wandering Assistance system (NAS) that assists older adults during night wandering episodes, decreases their anxiety and encourages them to sleep. The system was deployed in the homes of 78-year-old women for six weeks. The first step of the experimental protocol consists of completing different scales for profile identification: The Dementia Rating Scale (DRS), the Montreal Cognitive Assessment (MoCA), the Neuropsychiatric Inventory, the Geriatric Depression Scale, the Cornell Scale for Depression in Dementia and the 36-Item Short Form Health Survey. The second phase involves determining where to place the sensors and explaining the features of the system to the individual. The third phase involves installing 30 wireless sensors at various locations in the home and collecting data for 14 days. Diverse types of sensors were deployed: Contact door sensor, PIR sensor, pressure sensor, flow meter and power meter. Finally, the assistance phase was conducted considering the lifestyle of the monitored person. The assistance to the nocturnal wandering is ensured by lights and voice messages. The data analysis was performed by the K-means algorithm. The collected information helped the caregiver to obtain accurate information about the quality of the person’s night. But due to the small amount of data and the short duration of the experiment, it is not enough to accurately determine the impact of the assistance on the elderly person’s sleep.

Pinard et al. [61] present the Cognitive Orthosis for Cooking (COOK) project, which was designed to support meal preparation with and for people with severe Traumatic Brain Injury (TBI) and thereby improve their independence. It was implemented in the apartments of three participants. Their ages range from 39 to 57 and they had sustained severe TBI for over 10 years. The implementation began with teaching the participants how to use COOK in their apartments and ended with independent daily use of COOK. COOK is a web application installed on a tablet and is connected to a smart stove, which is equipped with various sensors: power sensors to identify which burner is on, infrared sensors to detect abnormal heat, oven door contact sensor and pressure sensor to identify objects placed on a burner. In addition, COOK is connected to a smart environment. Different sensors are installed in the house: PIR sensors, door contact sensors and flow meters. Two modules have been developed in the framework of this project: the Self-monitoring Security System (SSS) supervises the use of the stove (alerts and switches off the stove if a risky situation is detected) and the cognitive support module to support functional performance during meal preparation. After 6 months of use, two of the three users were very satisfied with the device.

Barsocchi et al. [56] propose an indoor localization technique related to the GiraffPlus European project. GiraffPlus is a long-term social interaction and monitoring project, installed in several test sites across Europe, to help people live independently. The sensors used in the test are the following: several PIR sensors installed in the main rooms, electrical usage sensors attached to the oven and personal computer, door usage sensors attached to the main door and finally a pressure sensor placed on the bed. The data gathered by these sensors was first filtered and then processed by the “where is” (WHIZ) algorithm. The result of the algorithm reflects the user’s routine, confirmed by comparison with user logs. In fact, the room-level tracking is a first step to associating a set of possible activities with the room where the activity is usually performed (cooking/kitchen, feeding/living room, bathing/bathroom, etc.). Experimental results show a sensitivity of 81%.

Lussier et al. [57] present a study commissioned by the Integrated Health and Social Services Centers (IHSSC) home care division of Montreal. Its aim was to develop an innovative technological approach to help assess and manage the risks associated with keeping elderly people at home who are at risk of self-neglect. The system developed has been installed for three home care recipients. The sensors used in the system are the following: (1) PIR sensors installed in various locations in the home, (2) magnetic contact sensors installed on the front door, frequently used drawer, fridge, utensil drawer, kitchen cabinet, and a food storage cabinet, and finally (3) smart electrical switches installed on the television and microwave. The system is capable of monitoring sleep, outings, inactivity, cooking-related activities and hygiene. Care professionals receive reports one month after the system is installed detailing the general routine of the elderly. These reports are considered by them to be reliable information that allows them to confirm or deny their hypothesis about the presence of risk (malnutrition) or to develop their intervention plan (no meal support). This information cannot be detected with their non-technological data collection (people at risk of self-neglect do not always provide reliable information for the questionnaires).

#### 6.1.3. Video and Audio Approaches

Seint et al. [64] propose a video monitoring system for medication intake and eating activity of the elderly. The system tags and tracks specific regions such as hands, head, and objects such as cups, and then extracts features that are inputs to the algorithm that identifies the activities. Finally, the system interprets medication intake using a hybrid PRNN-SVM (Pattern Recognition Neural Network) model and meal intake using rule-based learning and an occurrence counting method. A video camera was used in the experiment, and the classification rate of the drug-taking pattern is over 90% and over 95% for the meal-taking pattern when evaluated with 10 video sequences.

Cippitelli et al. [66] present a solution for automatic identification of eating and drinking actions of elderly people using the Kinect sensor, which provides color and depth streams, placed on the ceiling. The depth camera is used to transform the depth frame into 3D space. A body orientation algorithm is applied to identify the orientation of the person while sitting at the table. Then, the Self Organizing Map (SOM) algorithm models the upper part of the human body (head and hands) after filtering the point cloud. Finally, by merging the depth and RGB information in the same frame and mapping them, the system can evaluate a drink-taking action by analyzing the objects on the table such as the glass. Indeed, the raw depth frame does not discern the presence of dishes and glasses on the table. Each of the 35 young people performs the drinking action 1/2 times, generating a total number of 48 sequences. The algorithm classifies them correctly with a score of 98.3%.

Vuegen et al. [68] propose to identify ADLs of elderly people by using a wireless acoustic sensor network (WASN). Each sensor is composed of three linearly spaced microphones, and seven sensors were installed at different locations in the home environment for activity recording. Ten different activities were recorded in the living environment, and they were performed by two people several times. These activities are brushing teeth, washing dishes, dressing, eating, preparing food, setting the table, showering, sleeping, going to the bathroom and washing hands. Mel-Frequency Cepstral Coefficients (MFCCs), a well-known feature extraction approach in speech and speaker recognition applications, are extracted from the data, and then the SVM uses the normalized mean and variance of each MFCC dimension as features for ADL classification. The results indicate that the classification performance of WASN is 75.3 ± 4.3% on the clean acoustic data. In addition, artificial noise was created during the test and the classification performance of WASN under this condition is an absolute mean of 8.1% to 9.0% more accurate compared to the higher results obtained by the single microphone.

#### 6.1.4. Wearable Sensor Approach

Ueda et al. [70] propose a machine learning-based method for recognizing ADLs at home. It consists of using ultrasonic positioning sensors and power meters attached to the TV and stove. The ultrasonic positioning sensors consist of an ultrasonic transmitter called a TAG and receivers. The TAG is attached to the resident and the receivers are mounted on the ceiling of each room in the smart home in order to detect its position. The data used in this experiment is collected by two young men who lived in the smart home (experimental housing with one bedroom and one living room with kitchen built in the Nara Institute of Science and Technology, Japan) for three days each. The first step to identifying activities is to acquire the training data for machine learning. To easily obtain the training data, a recorded video is used as ground truth to label the sensor data according to the type of activity. The second step is to extract the feature value that is effective to identifying the activities and, finally, recognize the activities by employing the SVM algorithm. The method recognized six different activities (watching TV, taking a meal, cooking, reading a book, washing dishes and others) with an accuracy of about 85%.

Yunfei et al. [76] propose an approach for detecting ADLs via a smartphone. ADLs indoors are recognized by analyzing the combination of data from the audio, orientation of the heading of the phone, light level around the phone, Wi-Fi signals, GPS and other features such as the step detector. Audio-based recognition is done by matching raw audio features with the database of audio files that correspond to each category of activity (the sound of running water indicates specific acoustic information in the kitchen). Fingerprint-based localization is the technique used for the location indicator. In order to predict the location of the mobile, it is necessary to build a Wi-Fi fingerprinting database with Received Signal Strength Indicator (RSSI) data from several access points. Then, the location estimation is performed using SVM as a classifier. Results obtained in four apartments show that rates for the 6 ADLs (working on a desktop PC in the bedroom, walking, hygiene activities, cooking, washing dishes, and eating) are above 90%.

Tsang et al. [74] present ActiveLife, a system for tracking ADLs of patients with mild cognitive impairment (MCI) in their homes. A set consisting of three types of motion sensors is used in the system and placed on the thigh of one leg. The motion sensors are an accelerometer to measure linear acceleration, a gyroscope to measure angular acceleration and a magnetometer to measure magnetic field strength. The combination of the data collected by the sensors allows for the calculation of basic postures (standing, sitting and lying), transitional movement features and direction of the user and thus for activity recognition. The system focuses on the recognition of five indoor activities: sleeping, watching TV, going to the toilet, cooking and eating. All other activities, including outdoor activities, are classified as “other”. The activity classification algorithm consists of two steps. The first step is to classify the accelerometer and gyroscope data into transitions (walking movement) or activities (non-transition periods) using SVM. The second step is to use the SVM again to classify the basic activity posture. Then, by checking the direction and features of the transition motion, the algorithm can determine the current activity. The results show 99.8% accuracy in the classification of transitions and activities and 100% accuracy in the classification of different postures.

Park et al. [65] propose a method to recognize ADLs of the elderly using a combination of multi-view computer vision and radio-frequency identification (RFID)-based direct sensors. The vision system consists of two wide field-of-view (FOV) cameras and two narrow FOV cameras, all synchronized. The wide FOV cameras focus on displaying the person’s position in a 3D space, and the narrow FOV cameras focus on the detailed activities performed by the person in the kitchen. In addition, ADLs may involve multiple objects moving simultaneously, so a background model using K-means clustering was adopted. Additionally, to overcome the problem of multiple people in the smart home and identifying which one is performing an activity, a Probabilistic Appearance Model (PAM) that represents the color of people was used. The RFID system is composed of a wearable and short-range RFID reader (detection range of about 10–15 cm) and several RFID tags attached to various objects. When the person’s hand approaches an RFID-tagged object, the wristband detects it and transmits the information to the activity recognition system, and thus the system is able to learn object appearance patterns. The experiments were performed by five participants in the smart home testbed to recognize six activities: walking, sitting and watching TV, preparing a utensil, storing a utensil, preparing cereal and drinking water. Each person performs five repetitions per activity in two sessions. The result shows a mean accuracy of 83% in activity recognition.

As mentioned above, there are different approaches to detecting ADLs using several types of sensors. Each system has its advantages and drawbacks. Using a large number of sensors, as in the example of [62] which uses more than 80 sensors, complicates the installation process and increases the price of the system. In addition, video and audio approaches provide good insight into ADLs because they collect a large amount of information, but they remain the least preferred approach to identify ADLs due to privacy violation. Moreover, elderly users may not easily accept wearable sensors. Therefore, the binary and non-binary approach is the most widely used and preferred approach to detecting ADLs in the elderly.

### 6.2. Commercial Products

The miMonitor home monitoring system [78] allows families and care professionals to discreetly monitor and check relatives and patients in their homes. The system is composed of different sensors: motion sensors to monitor the movement inside the house, a contact door sensor to monitor the opening of the door, a smart plug to monitor the use of electrical appliances such as TV and kettle, and finally a camera to monitor the area where it is placed. All these sensors are connected via Wi-Fi and send alerts and notifications to the miMonitor mobile application, allowing, for example, to receive real-time notifications of events such as non-activity alerts, opening of the front door, use of plugged-in electrical appliances, etc.

Just checking [79] is an activity monitoring system that helps people with dementia live at home as long as possible, reassures families about their relatives and helps caregivers provide the right care at the right time. The system consists of five PIR sensors, two door contact sensors and a hub that has its own mobile connection. Data is sent from the sensors to the hub and then to the company’s servers. The user can access the system to view activity charts and receive notifications of problems.

The Canary Care system [80] allows older people to live in their homes longer and comfort their families. It consists of wireless sensors placed around the home to monitor various activities such as movement inside the house, bathroom movements, eating and drinking habits, medication intake and sleep. In addition, the system monitors the temperature inside the house. The system is composed of a PIR sensor, a door contact sensor, a visitor card and a hub. The visitor card is used to track visits to the home where the system is installed. For example, when the caregiver who regularly visits the elderly person swipes their card on the hub, the family can be notified and thus be reassured about the person monitored. The system allows viewing activity data, sets rules, and sends notification via SMS and email.

Kiwatch [81] proposes a remote monitoring system for the home care of elderly people. It uses different cameras installed in the main rooms (bedroom, kitchen, living room and in front of the main door) to reassure their families and make it easier for them to stay at home. With an integrated microphone and speaker, their families can chat with them at anytime and anywhere via their smartphone. In addition, the cameras are equipped with infrared LEDs for night vision to monitor the elderly in case of night wandering. Furthermore, the system can alert families in case of abnormal behavior, such as a fall and missing meals (an alert can be triggered when there is no movement in the kitchen at lunchtime).

Allovie [82] is a remote assistance system that ensures the safety of elderly people who choose to remain independent at home. It is composed of four devices allowing to trigger an alert in case of any problem: a medallion to be worn around the neck or a bracelet triggering an alert when the elderly person presses on it, a watch equipped with an automatic triggering system in case of a sudden fall, a call puller placed near the bed to call the center when the elderly person needs help, and finally four PIR sensors and a door contact sensor placed at strategic locations in the house allow to monitor the elderly person, to analyze their daily routine, and to send an alert in case of abnormal situation.

Rosie [83] is a reminder alarm clock designed to increase the independence and safety of the older people. It is a voice-activated memory aid and daily organizer. It is suitable for repeated tasks at specific times and days such as meals, medications, favorite activities, etc. It can contain 25 reminders with personalized voices such as those of family members for better compliance.

TruSense [84] is a smart home monitoring solution designed for the elderly. It consists of four motion sensors, a contact door sensor, a water sensor and the TruSense hub. The analysis of the data collected by the sensors allows to detect a variety of problems. Notification is sent when the elderly spends an unusually long time in a room which may indicate a fall or any other problems, when the elderly has left the house, when the person stays in bed longer than usual in the morning, when unsafe temperature for the person living in the house or a water leak in the bathroom or kitchen is detected, and, finally, a request for assistance from the TruSense’s 24-h emergency response team can be made.

Various commercial products have been proposed to monitor the change in behavior of elderly people in their daily living activities. These systems provide different benefits as they allow families and health care professionals to monitor and follow relatives and patients continuously in their homes. All these products, however, have different limitations. In fact, some commercial products focus on using cameras, such as [81], to monitor the elderly. But the elderly do not favor this method as we mentioned above. In addition, various products focus on basic alerts, such as non-activity alerts and opening the front door in the case of [78] or notification when the elderly spends an unusually long time in a room or when the elderly has left the house, for example, in the case of [84]. They do not use the collected data to identify more complicated ADLs such as eating, they are not able to identify the degradation of mobility and they do not use the data as ground truth to identify changes in behavior such as social isolation, loneliness, dementia, etc.

## 7. Discussion

In analyzing the findings of the review, current challenges and gaps that should be addressed in future research were identified and summarized as follows:

(1) Different kinds of sensors, ranging from PIR sensor to camera, were identified in the studies in order to recognize ADLs. Although there are promising results in recognizing them, it has not been possible to identify the best sensor for recognizing the meal-taking activity and mobility. A combination of different types of sensors usually provides better results in identifying the ADLs. In addition, intrusive sensors such as camera and microphone are not preferred in those types of monitoring systems due to their privacy violation.
(2) Different types of algorithms have been used to identify ADLs, but the majority of the algorithms use a supervised machine learning algorithm, which gives good results in detecting them. In every system, however, there is the problem of providing annotated data because self-annotation by the user causes different problems such as omissions, errors in entering certain labels, etc. In some systems, they used the camera as a tool to label ADLs, such as in the case of the system presented in [70]. However, we face the same problem of privacy violation of the monitored person despite using the camera only for labeling and not for identifying ADLs.
(3) The majority of data used in different systems are collected in smart home laboratory conditions during several days with participation of young adults. This first step is appreciable but it is not efficient. In fact, collecting real data in the homes of the persons is quite different compared to collecting the data in the laboratory because each person has its own rhythm when performing ADLs. They are not real enough, they do not allow enough variety and complexity and the observed person is more comfortable when performing tasks in their home. In addition, to ameliorate the results of identification of ADLs, systems need to collect data during several weeks and not for a few days because the rhythm of performing the activities can change. Furthermore, the purpose of the monitoring systems is to be installed in homes of seniors. Therefore, collecting and analyzing data of young adults is not adequate because the rhythm of realizing ADLs of the elderly is different from their rhythm. That is why collecting real data in homes of seniors for long period of time is the best way to obtaining better results in identifying ADLs.
(4) Despite much research in the field of ADL monitoring in the elderly, few researchers, such as [55], have used ADL identification as a first step to identifying potential risk of loneliness and depression in the elderly. We did not find any article, based on our research, which uses identification of ADLs (mobility and meal-taking activity) to identify seniors with risk of social isolation.

## 8. Conclusions

With advances in sensor technology such as miniaturization, wireless communication capabilities, reduced power consumption and affordability, ADL identification and overall awareness of personal health have been improved, including the ability to monitor elderly in their homes worldwide. Therefore, these systems meet the elderly’s desire to live as long as possible in their home and for their families to feel safe. More complex integrated sensor technologies, detection, and analysis algorithms will be developed in the coming years. The most important challenges are the development of a non-intrusive hardware implementation, electronic component efficiency, data analysis and interpretation, long-term monitoring, and acceptance by adults to install this system in their homes.

The objective of this paper is to provide an overview of the current status and future prospects of research and development in the field of monitoring systems focused on two main activities of daily living: meal-taking activity (shopping, cooking, eating and washing dishes) and mobility (inside the home and the act of going out). These two activities combined seem relevant for a prediction of risk of social isolation. With the new impact of the COVID-19 pandemic, the implementation of a continuous monitoring system is a solution to promote aging in place and prevent social isolation. After conducting this review for the period from 2010 to 2020, we found that little research has been done on monitoring these ADLs and social isolation. Even so, different systems have been proposed to identify ADLs using several types of sensors (wearable and non-wearable), and different algorithmic approaches (supervised or unsupervised learning, fuzzy logic, etc.). However, they raise different challenges related to user needs, privacy, system acceptance and performance of the proposed algorithms. In addition, the majority of articles limited their research on identifying ADLs. Different researchers use data collected in a laboratory environment by asking young participants to perform ADLs with advanced tasks. Despite the preferred approach of using sensors that respect the privacy of the monitored person, various systems have added a camera to label the collected data, such as [70]. Moreover, the use of a machine learning algorithm requires a large amount of collected data.

This paper addresses these issues and the different solutions reported in the literature and available on the market. There are two approaches to identifying social isolation of the elderly. The first is a self-reported survey designed for research purposes to assess social isolation. Despite the fact that the survey gives us an insight into the status of the elderly, they are considered outdated because they do not embrace new modes of communication like videoconferencing [3]. Also, the survey gives us a discontinuous observation on the status of the individual because the survey cannot be conducted frequently over a short period of time. Moreover, the surveys are declarative and therefore subjective. The second approach is technological monitoring. Indeed, advances in hardware and the use of different machine learning algorithms have improved ADL identification. Therefore, the system gives us a continuous observation on the status of the elderly, provides objective data and allows the collection of different relevant and useful information that could be related to social isolation. However, there are some limitations related to the monitoring system such as the use of intrusive or invasive sensors to identify ADLs such as cameras, microphones, patches, high system cost when using a large number of sensors, etc.

Finally, while the detection of ADLs seems to be an important step for the observation of the behavior of people at risk, it should be carried out by the least intrusive systems and with the help of new approaches enabled by machine learning. In addition, the analysis of the collected information, combining digital data with other data sources such as the health profile or the social environment of the person now makes it possible to consider the automatic prediction of risks. For example, we are currently working on the detection of social isolation through the analysis of activities related to mobility and eating [85].

## Figures and Tables

**Table 1:** Keywords used for the literature search.

Keywords	
Elderly people	Social isolation
Elderly monitoring	Loneliness
Smart home	Social Isolation in the elderly
Monitoring system	Limit social isolation
Health monitoring	Taking meals
User behavior	Monitor eating in elderly
Elderly People Living Alone	Eating Activity
Activity recognition	Non-intrusive sensor
Activity daily living	Unobtrusive sensors
Changing behavior in elderly	Wireless sensor
Monitor mobility in elderly	User privacy
Daily mobility	Smart sensing
Nutrition	Malnutrition

**Table 2:** Number of hits in the area of sleep monitoring systems research between 2010 and 2020.

Keywords	Web of Science	PubMed	IEEE Xplore
Monitor elderly	8700	120,291	2998
Elderly monitoring system	3545	22,742	2421
Monitoring technology	213,286	95,162	116,056
Long term elderly monitor	891	8858	205
Smart home	15,545	1101	13,497
Monitoring system	345,087	131,532	189,744
Health monitoring	256,174	177,388	31,965
Monitoring technology	213,286	95,162	116,056
User behavior	82,881	16,103	28,829
Elderly People Living Alone	877	4449	229
Activity recognition	56,194	49,094	12,687
Activity daily living	29,382	64,711	2589
Changing behavior in elderly	2333	91,998	117
Unobtrusive Activity Recognition	202	54	133
Mobility in smart house	140	12	62
Monitor mobility in elderly	372	1377	156
Daily mobility	8180	7926	1018
Daily mobility in elderly	1073	5135	86
Social isolation	17,026	17,947	538
Loneliness	10,501	5321	125
Social Isolation in the elderly	741	6711	42
Limit social isolation	1661	2144	39
Limit social isolation in elderly	71	850	1
Taking meals	4227	514	194
Monitor eating in elderly	66	1027	20
Eating Activity	16,859	18,186	346
Eating Activity in elderly	321	5266	21
Nutrition in elderly	10,593	9044	33
Malnutrition in elderly	2314	1893	6
Smart sensing	21,915	2694	13,830
Non-intrusive sensor	1186	173	898
Unobtrusive sensors	1296	529	1112
Wireless sensor	89,018	6251	116,392
User privacy	24,957	1109	18,278
User acceptance	17,489	4621	4199
User satisfaction	19,825	3240	6434
Elderly monitoring data processing	623	3860	576
Elderly monitoring big data	119	203	79
Elderly monitoring data mining	91	227	95
Elderly monitoring machine learning	252	747	273
Elderly monitoring artificial intelligence	177	1065	414

**Table 3:** Territories, solitudes; summary of key figures by territory [35].

	Metropolitan France	Rural Communities	Small Cities *	Medium Cities *	Large Provincial Cities *	Parisian Agglomeration
Occasional loneliness	27%	25%	31%	24%	28%	27%
Frequent loneliness	9%	7%	12%	8%	7%	10%
Leave home daily	60%	50%	57%	58%	64%	73%
Use public transport every week	18%	5%	5%	10%	28%	48%
Spend whole days without talking to anyone	19%	21%	20%	19%	18%	18%
Regularly invite each other to their homes	52%	56%	50%	53%	50%	49%
Lack of solidarity where they live	31%	27%	32%	30%	32%	37%

* Small cities: agglomerations of 2000 to 20,000 inhabitants. * Medium-sized cities: agglomerations of 20,000 to 100,000 inhabitants. * Large provincial cities: agglomerations of 100,000 to 200,000 inhabitants and more.

**Table 4:** Advantages and drawbacks of the sensors used by the authors in their work.

Sensor	Source	Position	Parameter Obtained	Advantages	Drawbacks
PIR	Huynh et al. [55]	PIR in every room	Detection of movement in each room	Low cost system Preserve privacy	Not able to distinguish who is moving if there is more than one person living in the same house
	Barsocchi et al. [56]	PIR in every room	Detection of movement in each room		
	Lussier et al. [57]	Two PIR sensors in the bedroom (one directed at the bed and another one towards the space between the exit and the bed) and one in the rest of each room	Detection of movement in different positions inside the house and the act of going out		
	Gochoo et al. [58]	31 PIR sensors in different locations around the house 4 Reed switch	Detection of movement in different positions inside the house		
	Dawadi et al. [59]	23 PIR sensors in different locations in the house	Detection of movement in different positions inside the house and the act of going out		
	Kenfack Ngankam et al. [60]	11 PIR sensors	Detection of movement in each room		
	Pinard et al. [61]	PIR sensors in each room and some around the stove	Detection of movement around the oven		
Reed switch	Huynh et al. [55]	Reed switch in outer door	Detection of the act of going out	Low cost system, preserve privacy	Not able to determine who is using the items if there is more than one person living in the same house
	Barsocchi et al. [56]	Reed switch in outer door, refrigerator and the door of the bedroom	Detection of the act of going out and the use of the refrigerator		
	Lussier et al. [57]	Reed switch in outer door, drawer, wardrobe, refrigerator, utensil drawer, kitchen cabinet, and food storage cabinet	Detection of the act of going out and the use of the drawer, wardrobe, the refrigerator, the utensil drawer, the kitchen cabinet, and the food storage cabinet		
	Gochoo et al. [58]	4 door sensors (back door, garage door, front door and pantry)	Detection of the act of going out and the use of the pantry		
	Dawadi et al. [59]	6 reed switches in different locations in the house	Detection of the act of going out and the use of different items in the kitchen		
	Kenfack Ngankam et al. [60]	13 Reed switch sensors in different locations in the house	Detection of the act of going out and the use of different items in the house		
	Pinard et al. [61]	2 reed switch sensors for oven door aperture and the outer door	Detection of the act of going out and the use of the oven		
	Pirzada et al. [62]	40 to 50 reed switch sensors in different locations in the house	Detection of the act of going out and the use of different items in the house		
Ultrasonic	Ghosh et al. [63]	The board fitted with 5 ultrasonic sensors has been suspended from the ceiling	Detection of body movements	Highly accurate sensing distance	
Video camera	Seint et al. [64]	Camera in front of the monitored person (tests realized in the laboratory)	Video of the monitored person	Provide rich information	Sensitive to light Do not preserve privacy
	Park et al. [65]	Two wide field-of-view (FOV) cameras and two narrow FOV cameras			
Kinect sensor	Cippitelli et al. [66]	Kinect sensor (RGB and depth camera) has been suspended from the ceiling	Color and depth streams	Provide rich information Robust to light variation	Do not preserve privacy
Depth sensor and thermal sensor	Zelun et al. [67]	Depth sensor and thermal sensor has been suspended from the ceiling	Depth and thermal streams	Robust to light variation	Expensive system
Pressure	Barsocchi et al. [56]	Pressure-sensitive mats in the bed and the chair	Presence of the person in the bed and chair	Easy to install Provide accurate information	Not able to determine who is moving if there is more than one person living in the same house
	Kenfack Ngankam et al. [60]	3 Pressure sensors: bed, sofa and chair	Presence of the person in the bed, sofa and chair		
	Pinard et al. [61]	4 Pressure sensors for 4 burners of the stove (to detect objects placed on burners)	Presence of objects placed on burners		
Microphone	Vuegen et al. [68]	7 nodes (each node composed of 3 microphones) in bedroom, bathroom, toilet, oven, table of the kitchen and 2 in the living room	Audio recording	Provide rich information	Do not preserve privacy
Flowmeters	Pinard et al. [61]	Flowmeters on the tap of the kitchen	The use of the kitchen faucet	Provide accurate information	Not able to determine who is moving if there is more than one person living in the same house Expensive
Float switch	Rebeen et al. [69]	Float switch in the toilet	Measure the toilet being flushed		
Wattmeter	Barsocchi et al. [56]	Wattmeter in the water boiler or oven	Using the water boiler or oven	Provide accurate information	Not able to determine who is moving if there is more than one person living in the same house
	Kenfack Ngankam et al. [60]	Wattmeter for the TV	The use of the TV		
	Pinard et al. [61]	4 Wattmeter for 4 burners of the stove	The use of the 4 burners of the stove		
	Ueda et al. [70]	Two Wattmeters for the TV and the cooking heaters	The use of the TV and the cooking heaters		
Power analyzer	Belley et al. [71]	Single power analyzer placed in the electric panel	The use of the TV and different electrical gadgets in the house (the 4 burners of the stove, the electric kettle, the oven, the toaster, the extractor hood, the coffee maker, the microwave oven, the hair dryer, the blender, the electric mixer, the stereo and the refrigerator)		Not able to determine who is moving if there is more than one person living in the same house Expensive sensor
	Fortin-Simard et al. [72]	Single power analyzer placed in the electric panel	The use of different electrical gadgets in the kitchen		
Passive RFID	Fortin-Simard et al. [73]	Antennas Tags in different items of the kitchen (each object has a specific size, a type so it is associated with one or many RFID tags)	The use of different items of the kitchen	Provide accurate information	Expensive Difficult to install due to the number of tags that need to be installed on different items
Ultrasonic positioning sensor	Ueda et al. [70]	19 receivers of the ultrasonic positioning sensor in different place in the house Ultrasonic positioning sensor attached to the body of the person	Location of the position of the person inside the house	Provide accurate information	Must be attached to the body Problem of battery duration
Active RFID	Park et al. [65]	Multiple RFID tags are attached to various objects including furniture, appliances, and utensils around the smart homes. Bracelet that contains the RFID reader	The use of different items of the kitchen	Provide accurate information	Must be attached to the body Problem of battery duration
Smartphone	Yunfei et al. [73]	Smartphone	Orientation of the phone head, light level around the phone, GPS and other functions such as step detector, accelerator and time stamp	Provide accurate information Easy to use Widespread between persons	Must be attached to the body Problem of battery duration Do not preserve privacy
Accelerometer	Tsang et al. [74]	Accelerometers	Body movement and posture	Low cost sensor	Must be attached to the body
	Charlon et al. [75]	Smart insole (contain accelerometer)			

**Table 5:** Combination of sensors used in different research works.

Source	PIR	Reed Switch	Ultrasonic Sensor	Camera	Pressure Sensor	Microphone	Electrical Power Sensors	Flowmeter	Float Switch	RFID	Smartphone	Accelerometer
Huynh et al. [55]	✓	✓										
Barsocchi et al. [56]	✓	✓			✓		✓					
Lussier et al. [57]	✓	✓					✓					
Gochoo et al. [58]	✓	✓										
Dawadi et al. [59]	✓	✓										
Kenfack Ngankam et al. [60]	✓	✓			✓		✓	✓				
Pinard et al. [61]	✓	✓			✓		✓	✓				
Pirzada et al. [62]		✓										
Ghosh et al. [63]			✓									
Seint et al. [64]				✓								
Park et al. [65]				✓						✓		
Cippitelli et al. [66]				✓								
Zelun et al. [67]				✓								
Vuegen et al. [68]						✓						
Rebeen et al. [69]	✓	✓							✓			
Ueda et al. [70]			✓				✓					
Belley et al. [71]							✓					
Fortin-Simard et al. [72]							✓			✓		
Fortin-Simard et al. [73]										✓		
Yunfei et al. [76]											✓	
Tsang et al. [74]												✓
Charlon et al. [75]												✓

**Table 6:** Combination of sensors used in different research works.

Source	Algorithms or Software Involved	Outputs	Evaluation Metrics (%)	Labelled Data
Barsocchi et al. [56]	(1) Data provided by the sensor filtered. In particular, data from the magnetic contacts and power usage sensors processed to obtain information about when they change their status. Moreover, the median filter applied to the spikes produced by the power usage sensor of the personal computer. (2) Room-level localization algorithm “where is” (WHIZ) exploits the data provided by the sensor in order to provide information about the location of the elderly. (3) A set of possible activities associated with the room where the activity is usually performed (cooking/kitchen, feeding/living room, etc.).	Detection of ADLs such as lunch/dinner, resting/pc/tv, sleeping and hygiene.	81% sensitivity	Yes
Lussier et al. [57]	(1) Algorithms developed to monitor sleep, going out for activities, low activity periods, cooking-related activities and hygiene-related activities. The algorithms built around assumptions about these different activities. (2) Codification and matrix building used for data analysis. First, descriptive codes created. These codes labeled units of text (words, sentences, paragraphs) that encompassed a distinct meaning with regard to how and why monitoring data was used by social and health care professionals. The coding grid emerged from the data. Second, matrices used to further analyze the decision-making process of the social and health care professionals.	Detection of ADLs. Results showed that AAL monitoring technologies provide health professionals with information about seniors related to self-neglect such as malnutrition, deficient hygiene, lack of household chores, oversleeping, and social isolation.	No data available	No data available
Gochoo et al. [58]	(1) The annotated binary data converted into a binary activity image for ADLs. (2) Activity images used for training and testing the Deep Convolutional Neural Network (DCNN) classifier. (3) Classifiers evaluated with 10-fold cross-validation method.	Detection of four ADLs: bed to toilet movement, eating, preparation meals, and relaxing. DCNN classifier gives an average accuracy of 99.36%.	99.36% accuracy	Yes
Dawadi et al. [59]	(1) Activity recognition based on SVM. (2) Support Vector Regression (SVR), Linear Regression (LR), Random Forest (RF) used to predict clinical scores of smart home residents using activity performance features computed from activity labeled sensor data.	Detection of seven ADLs: sleep, bed to toilet movement, cooking, eating, relaxation, personal hygiene, and the mobility of the resident inside the home. There is a correlation between the predicted clinical assessment using activity behavior and the mobility scores provided by the clinician.	95% accuracy	Yes
Pirzada et al. [60]	(1) The K-Nearest Neighbors algorithm (KNN) used to detect any irregular activity. In addition, the training and test data use the k-fold technique to generate different sets in the iteration.	Detection of anomalies in patterns.	No data available	No data available
Ghosh et al. [63]	(1) Support Vector Machine (SVM) with linear kernel, K-Nearest Neighbors (KNN) and decision tree techniques used on ultrasonic sensors data.	Detection of standing, sitting and falling.	90% accuracy	Yes
Rebeen et al. [69]	(1) The sequence of binary sensor features with incremental fuzzy time windows (FTWs) extracted, equal size (1 min) temporal windows (ESTWs) and Raw Last sensor Activation (RLA) in one-minute windows. (2) ADLs identified using different machine learning algorithm: Long Short-Term Memory (LSTM), Convolutional Neural Network (CNN) with ESTWs, C4.5 and SVM with RLA and LSTM, CNN and hybrid CNN LSTM model with FTWs.	Better results in recognition activities (eating, grooming, going out, showering, sleeping, saving time and going to the bathroom) when the recognition of the activity is delayed, preceding 1-min sensor activations with 5-min delays (20-min delay, 1-h delay, etc.) compared to considering only the 1-min delay sensor data.	CNN LSTM: 96.97% and 96.72% f1-score for the first and second database, respectively	Yes
Seint et al. [64]	(1) Labeling the colored bottles by RGB color space and labeling the skin parts by YCbCr color space, then tracking the desired objects. (2) Features were extracted for the drug intake model and the dietary activity model. (3) Hybrid PRNN-SVM (Pattern Recognition Neural Network) model was used for classification and interpretation of drug intake activity. (4) Rule-based learning with occurrence count method was used for classification and interpretation of meal intake activity.	Detection of medication and meal intake.	90% accuracy for taking medication and 95% accuracy for taking meals	Yes
Cippitelli et al. [66]	(1) A body orientation algorithm applied to the depth frame to identify the orientation of the person while sitting to the table. Then, point cloud filtering and Self-Organizing Map (SOM) algorithm applied for the upper part of the human body. (2) With subsequent mapping, depth and RGB information are combined in the same frame.	Detection of eating and drinking actions.	98.3% accuracy	Yes
Vuegen et al. [68]	(1) Feature extraction from acoustic sensor data performed using Mel-Frequency Cepstral Coefficients (MFCCs) approach. (2) Support Vector Machine (SVM) used for ADL classification.	Detection of brushing teeth, washing dishes, dressing, eating, preparing food, setting table, showering, sleeping, toileting and washing hands.	78.6 ± 1.4% accuracy	Yes
Yunfei et al. [72]	(1) The mobile device’s orientation is detected by the GPS sensor. (2) A Wi-Fi fingerprinting database created using data from multiple locations inside the house’s Received Signal Strength Indicator (RSSI). Then, SVM was used as classifier to conduct location estimation. (3) The sounds were categorized using timbres	Detection of 6 ADLs: working on a desktop PC in the bedroom, wandering walk, hygiene activities, cooking, washing dishes, and eating.	Between 92.35% and 99.17% accuracy for each of the four databases	Yes
Tsang et al. [74]	(1) Using SVM, the accelerometer and gyroscope data were classified into transitions (walking motion) or activity (non-transition periods). (2) The activity’s basic posture is classified by SVM. Then, the direction and features of the transition motion were examined to determine the current activity.	Recognition of five indoor activities: sleeping, watching TV, toileting, cooking and eating. All other activities including outdoor activities are assigned to “others”.	99.8% accuracy	Yes
Park et al. [65]	(1) Homography mapping for 3D localization of people from the two wide-FOV cameras and foreground segmentation for unoccluded views of people used for (fine) body-level analysis from the two narrow-FOV cameras. K-means clustering adopted for the background model and the probabilistic appearance model to identify the person performing an activity.	Recognition of six activities: walking around, sitting and watching TV, preparing a utensil, storing utensil, preparing cereal and drinking water.	83% mean accuracy for all activities	Yes
Ueda et al. [70]	(1) The feature value of the sensor data extracted from the 5-min time interval that is labeled (a recorded video is used as ground truth to label the sensor data according to the type of activity). (2) SVM used to identify the activities using the feature values from the sensor data.	Recognition of six different activities: watching TV, taking a meal, cooking, reading a book, washing dishes, and others.	85% accuracy	Yes

**Table 7:** Participants, duration and location of data collection.

Source	Number of Participants	Duration of Data Collection	Gender	Age	Health Status of the Participants	Type of Home Where Test Was Done
Huynh et al. [55]	43	6 months	19 males, 24 females	Mean age 77.59 and standard deviation 7.65	No data available	Apartment
Barsocchi et al. [56]	1	10 days	One female	No data available	No data available	GIRAFFPLUS test site
Lussier et al. [57]	3	1 month	One female, two males	(1) 91-year-old woman (2) 49-year-old man (3) 87-year-old man	Numerous health issues for each person	(1) Care recipients’ homes (2) Low-rent housing unit (3) Residence for senior
Gochoo et al. [58]	1	21 months	One female	No data available	Healthy person,	Laboratory Smart home
Dawadi et al. [59]	18	2 years	5 females, 13 males	(M = 84.71, SD = 5.24, range 73−92)	cognitively healthy (N = 7), at risk for cognitive difficulties (N = 6), and experiencing cognitive difficulties (N = 5)	Laboratory Smart home
Kenfack Ngankam et al. [60]	1	6 weeks	One female	78-year-old	Moderate cognitive impairment	Apartment
Pinard et al. [61]	3	6 months	Three males	Ages range from 39 to 57	Sustained severe traumatic brain injury	Individual apartment in the supported-living residence
Pirzada et al. [62]	2	14 days	No data available	No data available	No data available	Apartment
Ghosh et al. [63]	10	100 samples for each	No data available	No data available	No data available	Laboratory Smart home
Seint et al. [64]	Different persons	10 sequences	No data available	No data available	No data available	Laboratory Smart home
Park et al. [65]	5	Each person does five repetitions per activity in two sessions	No data available	No data available	No data available	Laboratory Smart home
Cippitelli et al. [66]	35	48 sequences	No data available	22 − 38 years	No data available	Laboratory Smart home
Vuegen et al. [68]	2	Multiple samples of 10 different activities	No data available	No data available	No data available	Apartment
Rebeen et al. [69]	2	(1) 14 days (2) 22 days	No data available	No data available	No data available	Apartment
Ueda et al. [70]	2	3 days	One male	In their twenties	No data available	Laboratory Smart home
Belley et al. [71]	1	10 consecutive tests for different selected sequences of tasks	No data available	No data available	No data available	Laboratory Smart home
Fortin-Simard et al. [72]	1	Five different ADLs performed 25 times	No data available	No data available	No data available	Laboratory Smart home
Fortin-Simard et al. [73]	1	Five different ADLs performed 25 times	No data available	No data available	No data available	Laboratory Smart home
Yunfei et al. [76]	4	No data available	No data available	No data available	No data available	Apartment
Tsang et al. [74]	1	Different samples	No data available	No data available	No data available	Apartment
Charlon et al. [75]	9	Use of smart insole for half hour for each participant	Six males, three females	Mean age was 70.1 years (65 to 75)	healthy	Laboratory Smart home

## Data Availability

Not applicable.
